# Vertebral Artery Stenoses Contribute to the Development of Diffuse Plaques in the Basilar Artery

**DOI:** 10.3389/fbioe.2020.00168

**Published:** 2020-03-06

**Authors:** Yundi Feng, Jian Liu, Tingting Fan, Wenxi Zhang, Xiaoping Yin, Yajun E, Wenchang Tan, Yunlong Huo

**Affiliations:** ^1^Department of Mechanics and Engineering Science, College of Engineering, Peking University, Beijing, China; ^2^PKU-HKUST Shenzhen-Hongkong Institution, Shenzhen, China; ^3^Department of Cardiology, Peking University People's Hospital, Beijing, China; ^4^Department of Radiology, Affiliated Hospital of Hebei University, Hebei University, Baoding, China; ^5^Department of Neurology, Affiliated Hospital of Hebei University, Hebei University, Baoding, China; ^6^Shenzhen Graduate School, Peking University, Shenzhen, China; ^7^Institute of Mechanobiology & Medical Engineering, School of Life Sciences & Biotechnology, Shanghai Jiao Tong University, Shanghai, China

**Keywords:** posterior circulation, vertebrobasilar disease, diffuse artery disease, stenosis, computed tomography angiography

## Abstract

Vertebral artery (VA) stenosis is relevant to a high early risk of recurrent stroke and basilar artery (BA) is the most common intracranial site of atherosclerotic lesions. It is important to show predictive risk factors for transient ischemic attack (TIA) or posterior infarctions. The aim of the study is to investigate morphometry and hemodynamics in intracranial vertebral and basilar arteries of health and diseased patients to enhance the risk assessment. Based on the geometrical model reconstructed from CTA images in 343 patients, a transient three-dimensional computational model was used to determine the hemodynamics. Patients were classified in symmetric, asymmetric, hypoplastic, and stenotic groups while patients in the stenotic group were divided into unilateral, bilateral, bifurcation, and tandem stenotic sub-groups. Patients in bilateral, bifurcation, and tandem stenotic sub-groups had significantly lower basilar artery diameters than other groups. Patients in the stenotic group had significantly higher surface area ratio (SAR) of high time-averaged wall shear stress gradient (TAWSSG) and higher incidence of TIAs or posterior infarctions than other groups while patients in the tandem stenotic sub-group had the highest values (SAR-TAWSSG of 57 ± 22% and TIAs or posterior infarction incidence of 54%). The high SAR-TAWSSG is predisposed to induce TIAs or posterior infarction.

## Introduction

There is higher prevalence of intracranial vertebrobasilar atherosclerosis in Asians than Caucasians (Caplan et al., 2004; Lee et al., 2006; Nouh et al., 2014). The high-grade artery stenosis can induce hypoperfusion in patient vertebrobasilar system. Intra-arterial embolism due to intracranial plaque rupture is a more common mechanism of transient ischemic attacks (TIAs) and posterior ischemic strokes than hypoperfusion in Asians (Caplan et al., 2005a,b; Savitz and Caplan, 2005; Markus et al., 2013; Qureshi and Caplan, 2014; Ritz et al., 2014). Vertebral artery (VA) stenosis is relevant to a high early risk of recurrent stroke while basilar artery (BA) is the most common intracranial site of atherosclerotic lesions (Mattle et al., 2011; Markus et al., 2013; Qureshi and Caplan, 2014). There is, however, lack of comprehensively morphometric analysis to show the relationship between vertebral artery stenoses and basilar artery lesions.

There are various potential risk factors for the development of intracranial diseases, e.g., hypertension, smoking, hyperhomocysteinemia (HHcy), dyslipidemia, diabetes mellitus (Arbab-Zadeh and Fuster, 2015; Toutouzas et al., 2015), and biomechanical stresses (Chatzizisis et al., 2008; Seneviratne et al., 2013; Toutouzas et al., 2015; Thondapu et al., 2016). Previous studies have shown that a mild stenosis significantly increased the time-averaged wall shear stress (TAWSS) and decreased the oscillatory shear index (OSI) to result in a more uniform distribution of hemodynamic parameters inside the stenosis (Huo et al., 2013a; Huang et al., 2016). The stenosis also created the disturbed flows distal to the stenosis. In the intracranial vertebrobasilar arteries, the upstream stenoses in vertebral arteries or flow confluence (left and right vertebral arteries merging into the basilar artery) can significantly affect the hemodynamics in the distal basilar artery. The mild diseases in the downstream basilar artery has negligible hemodynamic effects on the upstream vertebral artery (Chen et al., 2016). Hence, we hypothesize that two-sided vertebral artery stenoses (including a stenosis at each vertebral artery and multiple stenoses in both vertebral arteries) or bifurcation stenoses at the flow confluence can be risk factors to accelerate the development of diffuse basilar artery lesions (i.e., the widespread disease in the basilar artery that can increase plaque burdens).

The objective of the study is to investigate morphometry and hemodynamics in intracranial vertebrobasilar arteries of healthy and diseased patients as well as the relationship between vascular diseases and TIA/posterior ischemic events. Here, three-dimensional (3D) geometrical models of intracranial vertebrobasilar arteries were reconstructed from CTA (i.e., Computer Tomography Angiography) images in 343 patients. Based on the geometrical model, a transient 3D finite volume model (FVM) was used to solve the continuity and Navier-Stokes equations to compute the flow fields, from which multiple hemodynamic parameters (e.g., TAWSS, OSI, and TAWSS gradient-TAWSSG) were obtained. The significance, implication, and limitation of the study are discussed to aid in clinical decision making.

## Materials and Methods

### Study Design

This retrospective study assessed morphometry and hemodynamics of the intracranial vertebrobasilar system in 343 patients (196 men and 147 women; mean age 56 ± 12 years), who underwent cerebral CTAs at the Affiliated Hospital of Hebei University, China, from January 2014 to May, 2017. The study was approved by the Institutional Review Board (IRB) at the Affiliated Hospital of Hebei University, China, which conformed the declaration of Helsinki and Good Clinical Practice Guidelines of National Medical Products Administration of China.

Based on the morphometry of the intracranial vertebrobasilar system, patients are classified in four groups: (1) the two vertebral arteries are normal and have similar diameters (i.e., symmetric group); (2) the diameter in an entire vertebral artery is confined to be <3 mm or the ratio of one vertebral artery with lower mean diameter value to the other with higher value is 1:1.7 or more (Touboul et al., 1986; Trattnig et al., 1991) (i.e., hypoplastic group); (3) except for the hypoplastic group, the diameter difference of two vertebral arteries is no lower than 3 mm or the vertebral artery connected to the basilar artery in a more straight fashion (i.e., the degree between the center lines of basilar artery and vertebral artery in the bifurcation area is closer to 180°) if the two vertebral arteries have similar diameters (Hong et al., 2009) (i.e., asymmetric group); and (4) there is at least an area stenosis ≥70% in vertebral arteries or basilar artery (i.e., stenotic group), where the stenotic degree is determined using the method in a previous study (Samuels et al., 2000). There are stenoses of multiple degrees at various sites of the intracranial vertebrobasilar system, i.e., left vertebral artery, right vertebral artery, basilar artery, and bifurcation of flow confluence (left and right vertebral arteries merging into basilar artery). Furthermore, patients in the stenotic group are classified in four sub-groups: (1) a stenosis in unilateral vertebral artery (i.e., unilateral sub-group), (2) a stenosis at both vertebral arteries (i.e., bilateral sub-group), (3) stenoses at the bifurcation of flow confluence (i.e., bifurcation sub-group), and (4) multiple stenoses at various sites of the intracranial vertebrobasilar system (i.e., tandem sub-group). Based on the cardiovascular disease risk factors, patients are classified as hypertension, diabetes mellitus, smoking, and dyslipidemia groups, where a patient with multiple diseases is classified to different groups.

### Study Population

A total of 343 patients were identified from a large database at the Affiliated Hospital of Hebei University if they had none of the following symptoms: (1) rhythm other than sinus; (2) contraindication to iodinated contrast agents; (3) end stage renal disease requiring dialysis; and (4) previously surgery or artificial device in the vertebrobasilar system. The body mass index (BMI), systolic and diastolic blood pressures, cholesterol, triglycerides, low-density lipoprotein (LDL), high-density lipoprotein (HDL), and fasting glucose were collected by routine physical examination. These patients underwent cerebral CTAs to evaluate whether they have TIA or stroke (Savitz and Caplan, 2005).

### CTA Imaging Acquisition

Similar to previous studies (Huang et al., 2016; Yin et al., 2016), all patients underwent CTA scanning from the aortic arch to the vertex using a 64-slice multidetector CT scanner (SOMATOM Sensation 64, SIEMENS). Briefly, non-enhanced CT brain scan was performed first followed by the contrast enhanced CTA. CTA images were acquired when 80 ml contrast agent (Omnipaque 350, Amersham Health) was injected at a rate of 4 ml/s followed by IV injection of saline chase of 30 ml at a rate of 2 ml/s. A bolus tracking method (Smart Prep) was used to monitor the optimal contrast enhancement (Mnyusiwalla et al., 2009). Study parameters included scan time of 4.5 s, caudo-cranial scan direction, 120 kV and 200 mAs radiation parameters, rotation time of 0.5 s, slice collimation of 0.6 mm, slice width of 0.75 mm, 1.0 pitch, 0.4 mm reconstruction increment and kernel of H30f.

### CTA Imaging Analysis

**Figure 1:**
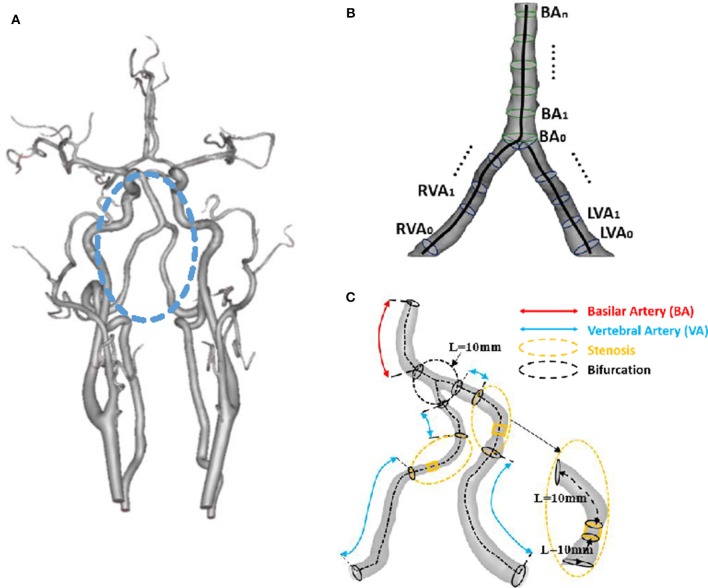
**(A)** 3D geometrical model of cerebral arteries reconstructed from a patient CTA (The vertebrobasilar system inside the circle is comprised of left and right vertebral arteries and basilar artery); **(B)** Schematic representative of the best fit diameter D_fit_ along centerlines of left and right vertebral arteries and basilar artery, where the best fit diameter is computed as twice the average radius between the point on the centerline and the contour forming the contour of the 3D object; and **(C)** Schematic representative of bifurcation and stenotic regions for determination of morphometric and hemodynamic parameters.

The 3D geometry and morphometry of vertebrobasilar arteries, as shown in Figure 1, were extracted from patient CTA images of symmetric, asymmetric, hypoplastic and stenotic groups using the MIMICS software (Materialize, NV, Belgium) (Yin et al., 2016). We focused on the intracranial vertebrobasilar bifurcation that is comprised of vertebral arteries and basilar artery. Here, the morphometric analysis only included the intracranial portion of vertebral arteries [i.e., the V4 segment (Caplan et al., 2004)] and the entire basilar artery down to the distal bifurcation (i.e., basilar artery bifurcating into the right and left posterior cerebral arteries-PCAs). In the MIMICS software, a centerline was formed by a series of center points which was located in the center on the cross–sectional views of the contour of the 3D vessel. Subsequently, the best fit diameter, D_fit_, was calculated through a least-squares best-fit circle based on points on the perimeter of the cross-sectional area. The CTA reconstruction and imaging analysis were performed by three researchers at Peking University as well as a Radiologist and a Neurologist at the Affiliated Hospital of Hebei University. The reproducibility of those measurements from the two universities showed κ value approximately equal to 0.86 (Cohen, 1986).

### Hemodynamic Analysis

Similar to previous studies (Huang et al., 2016; Yin et al., 2016), computational fluid dynamic (CFD) simulations were performed to analyze the flow patterns in the intracranial vertebrobasilar bifurcation reconstructed from patient CTA images. Briefly, based on the morphometric data, geometrical models were created in the Geomagic Studio software (3D Systems, Rock Hill, USA), which were meshed using the ANSYS ICEM (ANSYS Inc., Canonsburg, USA). The Navier-Stokes and continuity equations were solved to compute the distribution of pressure and flow in the ANSYS FLUENT (ANSYS Inc., Canonsburg, USA). The aortic pressure wave obtained from a previous study (Yin et al., 2016) was normalized by the time-averaged value and then scaled back to the physiological range based on the systolic and diastolic pressures measured in each patient. The scaled pressure wave was set as the boundary condition at the inlet of left and right vertebral arteries (i.e., the inlet arising from the subclavian arteries). The resistance boundary condition was set at each outlet, based on intraspecific scaling laws of vascular trees derived theoretically and validated experimentally in various organs and species (Huo and Kassab, 2012) including the cerebral circulation (Cassot et al., 2009), similar to a previous study (Yin et al., 2016).

A total of ~200,000–500,000 tetrahedral shaped elements with boundary layers for accurate resolution (element size = 0.3 mm, layers = 3, and height ratio = 1.5) were necessary to accurately mesh the computational domain. Three cardiac cycles were required to achieve the convergence for the transient analysis. We have recently shown negligible difference of hemodynamic parameters between Newtonian and non-Newtonian (i.e., Carreau fluid) models (Yin et al., 2016). Hence, viscosity (μ) and density (ρ) of the solution were assumed as 4.5 × 10^−3^ Pa·s and 1,060 kg/m^3^, respectively, to mimic the blood flow with a hematocrit of about 45% in these arteries. Hemodynamic parameters, TAWSS, OSI, and TAWSSG, were determined from the computed flow fields. We further computed SAR-TAWSS (Malek et al., 1999), SAR-OSI (Nordgaard et al., 2010; Huo et al., 2013a), and SAR-TAWSSG (Kleinstreuer et al., 2001; Fan et al., 2016) within the vertebral arteries, bifurcation of flow confluence, and basilar artery (see the nomenclature).

### Statistical Analysis

All data analysis was performed in blinded fashion. The mean ± SD (standard deviation) of morphometric and hemodynamic parameters were computed by averaging over all subjects in each group. The unpaired student *t*-test (GraphPad Prism 5 software) was used to compare demographic parameters among symmetric, asymmetric, hypoplastic, and stenotic groups as well as unilateral, bilateral, bifurcation, and tandem stenotic sub-groups, where *p* < 0.05 represented statistical difference. Two-way analysis of variance (SPSS 20 software) was selected to test the difference of basilar artery lumen diameters in two different ways, i.e., demographic parameters (hypertension, diabetes mellitus, smoking, and dyslipidemia) and morphometric variables (symmetric, asymmetric, hypoplastic, and stenotic vertebral arteries). The conditional forward binary logistic regression (two tailed and α of 0.05) (SPSS 20 software) was demonstrated to determine which variables are better predictors of diffuse basilar artery lesions as well as TIAs or posterior infarctions, where *p* < 0.001 represented statistical difference, similar to a previous study (Gong et al., 2016).

To illustrate the difference of hemodynamic parameters between normal patients and patients with TIAs or posterior infarctions, we randomly selected 13, 20, and 5 patients from the symmetric, asymmetric and hypoplastic groups, respectively, who have no TIAs and posterior infarctions. The patients were ~20% of patients with no TIAs and posterior infarctions in each group. Accordingly, we selected all patients with TIAs or posterior infarctions from unilateral, bilateral, bifurcation, and tandem stenotic sub-groups. The mean ± SD of hemodynamic parameters were computed by averaging over those subjects.

## Results

Table 1 summarizes patient demographics in symmetric, asymmetric, hypoplastic, and stenotic groups at the age of 55 ± 14, 55 ± 11, 57 ± 12, and 59 ± 12. Patients in the stenotic group have higher incidence of hypertension, diabetes mellitus, and HHcy than those in other groups. Patients in hypoplastic and stenotic groups have significantly higher incidence of posterior infarctions than those in other groups. Patients in the stenotic group have the highest incidence of TIAs.

**Table 1:** Baseline characteristics of the study population and morphometry of intracranial vertebrobasilar arteries in symmetric, asymmetric, hypoplastic, and stenotic groups.

	**Symmetric**	**Asymmetric**	**Hypoplastic**	**Stenotic**	***P* < 0.05**
N	74	127	42	100	N/A
Male, *N* (%)	47 (63.5)	61 (48.0)	23 (54.8)	65 (65.0)	N/A
Age, year	55 ± 14	55 ± 11	57± 12	59 ± 12	(Asym-Sten)
BMI	25.6 ± 3.4	25.1 ± 3.7	26.0 ±3.8	26.8 ±3.1	(Asym-Sten)
Hypertension, *N* (%)	45 (60.8)	88 (69.3)	29 (69.0)	74 (74.0)	N/A
Diabetes mellitus, *N* (%)	16 (21.6)	25 (19.7)	8 (19.0)	33 (33.0)	N/A
Active smoker, *N* (%)	33 (44.6)	48 (37.8)	21 (50.0)	35 (35.0)	N/A
Hyperlipemia, *N* (%)	11 (14.9)	27 (21.3)	8 (19.0)	16 (16.0)	N/A
TIA, *N* (%)	I (1.4)	7 (5.5)	4 (9.5)	23 (23.0)	N/A
HHcy, *N* (%)	13 (17.6)	14 (3.2)	10 (23. 8)	27 (27.0)	N/A
Posterior infarction, *N* (%	9 (12.2)	15 (11.8)	12 (28.6)	27 (27.0)	N/A
**Blood pressure, mmHg**					
Systolic P	151 ±21	145 ± 23	146 ± 22	151 ±23	None
Diastolic P	87± 17	89 ± 1 5	87 ± 13	89 ± 12	None
ΔP	64 ± 18	56 ± 16	60 ± 17	63 ± 18	(Asym-Sym) (Asym-Sten)
LDL, mmol/l	2.88± 0.69	2.72± 0.72	2.72± 0.71	2.73 ± 0.75	None
HDL, mmol/l	1.06 ± 0.22	1.07± 0.28	0.98± 0.22	0.97 ± 0.23	(Sym-Sten) (Asym-Sten)
LDL/HDL	2.78± 0.69	2.65 ± 0.82	2.81 ± 0.66	2.89 ± 0.85	None
Fasting glucose, mmol/l	6.33 ± 1.93	6.18± 2.06	6.91 ±2.80	6.77 ± 2.68	None
Total cholesterol, mmol/l	4.74 ± 0.90	4.61 ±0.95	4.41 ± 0.94	4.43± 1.02	(Sym-Sten)
Triglycerides, mmol/l	1.63 ± 0.89	1.76 ± 1.30	1.68± 1.09	1.73± 0.83	None
Dominant VA, mm	3.61 ± 0.49	3.98± 0.52	4.02 ± 0.65	3.89 ± 0.84	All except for (Sym-Sten) (Asym-Hypo)
Affiliate VA, mm	3.61 ± 0.49	3.16 ± 0.49	2.27 ± 0.38	2.92 ± 0.81	All
Basilar artery, m m	3.93 ± 0.45	3.97± 0.54	3.66 ± 0.64	3.61 ± 0.49	All except for (Asym-Sym)

**Figure 2:**
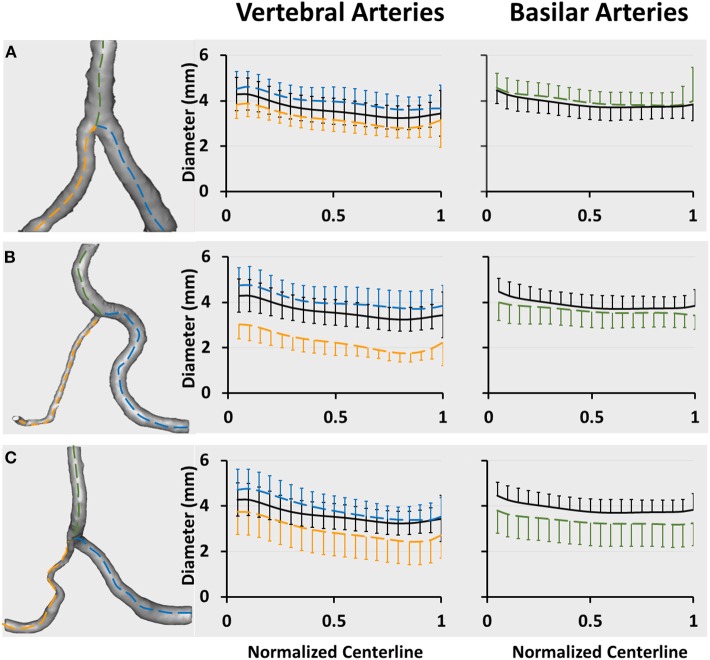
Vertebrobasilar arteries reconstructed from patient CTA images and a plot of D_fit_ along normalized vertebral artery and basilar artery centerlines in **(A)** asymmetric, **(B)** hypoplastic, and **(C)** stenotic groups. The vertebral artery centerlines are normalized by the accumulative length from the upstream inlet of left and right vertebral arteries (i.e., the centers of LVA_0_ and RVA_0_ in Figure 1B) to the inlet of basilar artery (i.e., BA_0_ in Figure 1B). The basilar artery centerline is normalized by the accumulative length from the inlet of basilar artery (i.e., BA_0_ in Figure 1B) to the flow outlet of basilar artery (i.e., BA_n_ in Figure 1B). The black line refers to the mean value of D_fit_ (averaged over 74 patients) in the symmetric group. The blue and yellow lines refer to the mean value of D_fit_ along normalized vertebral arteries of large and small sizes, respectively. The green line refers to the mean value of D_fit_ along the normalized basilar artery. The error bars refer to the standard deviation.

Figures 2A–C show a plot of D_fit_ along normalized vertebral artery and basilar artery centerlines in asymmetric, hypoplastic, and stenotic groups as compared with that in the symmetric group. The black line refers to the mean value of D_fit_ (averaged over 74 patients) in the symmetric group. The blue and yellow lines refer to the mean value of D_fit_ along the normalized vertebral arteries of the three groups, which are larger and smaller, respectively, than that in the symmetric group. The green line refers to the mean value of D_fit_ along the normalized basilar artery of the three groups. The error bars refer to the standard deviation. Table 1 also lists mean ± SD diameters of vertebral arteries and basilar artery in the four groups. The lumen size shows no statistical difference along the normalized basilar artery of hypoplastic, asymmetric, and symmetric groups. The lumen size along the normalized basilar artery of the symmetric group is significantly lower (*p* < 0.05) than other groups.

**Figure 3:**
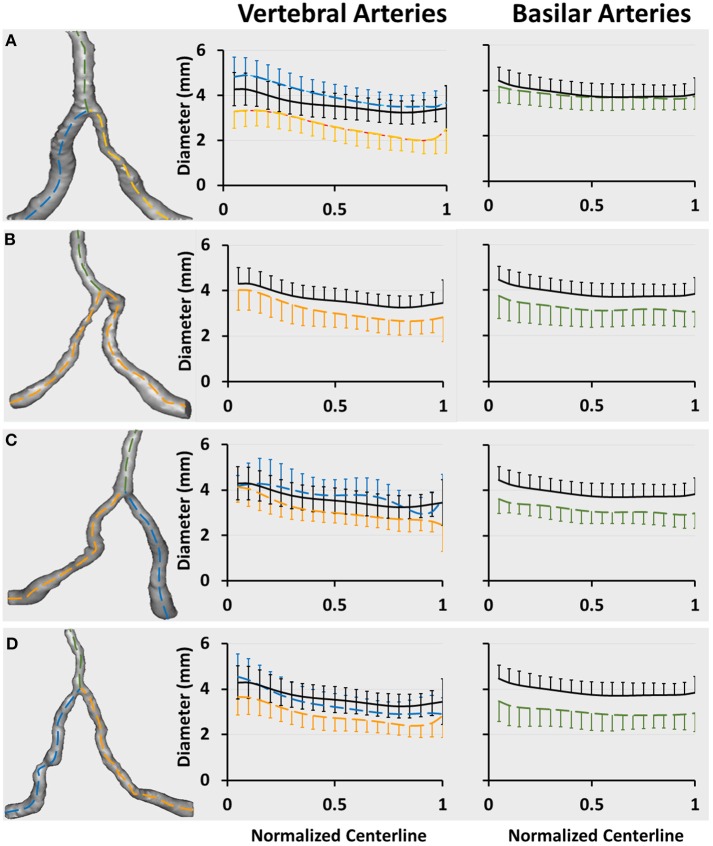
Vertebrobasilar arteries reconstructed from patient CTA images in the stenotic group and a plot of D_fit_ along normalized vertebral artery and basilar artery centerlines in **(A)** unilateral (31 patients), **(B)** bilateral (21 patients), **(C)** bifurcation (11 patients), and **(D)** tandem (37 patients) stenotic sub-groups. The black line refers to the mean value of D_fit_ (averaged over 74 patients) in the symmetric group. The blue and yellow lines in **Figures A,C,D** refer to the mean value of D_fit_ along normalized vertebral arteries of large and small sizes, respectively. The yellow line in **Figure B** refer to the mean value of D_fit_ along the normalized vertebral artery given similar sizes between left and right vertebral arteries. The green line refers to the mean value of D_fit_ along the normalized basilar artery. The error bars refer to the standard deviation.

Figures 3A–D show a plot of D_fit_ along normalized vertebral artery and basilar artery centerlines in unilateral, bilateral, bifurcation, and tandem stenotic sub-groups. The lumen size along the normalized basilar artery in bilateral, bifurcation, and tandem stenotic sub-groups is significantly lower than that in the symmetric group despite similar sizes between the unilateral stenotic sub-group and symmetric group. The conditional forward binary logistic regression shows that two-sided vertebral artery stenoses (Wald test = 21.08, *p* < 0.001) or bifurcation stenoses (Wald test = 12.22, *p* < 0.001) are better risk factors for the diffuse reduction of basilar artery lumen size than the symmetric (Wald test = 1.76, *p* = 0.82), asymmetric (Wald test = 5.08, *p* = 0.24) and hypoplastic vertebral arteries (Wald test = 6.29, *p* = 0.14) as well as the unilateral stenosis (Wald test = 1.22, *p* = 0.27).

There are 11, 8, 3, and 20 patients with TIA or posterior infarction events in unilateral, bilateral, bifurcation, and tandem stenotic sub-groups including a total of 31, 21, 11, and 37 patients, respectively. The incidence of TIAs or posterior infarctions is significantly higher than that in symmetric and asymmetric groups in Table 1. Patients in the hypoplastic group show similar incidence as those in the unilateral stenotic sub-group, but have significantly lower incidence than that in the tandem stenotic sub-group.

**Figure 4:**
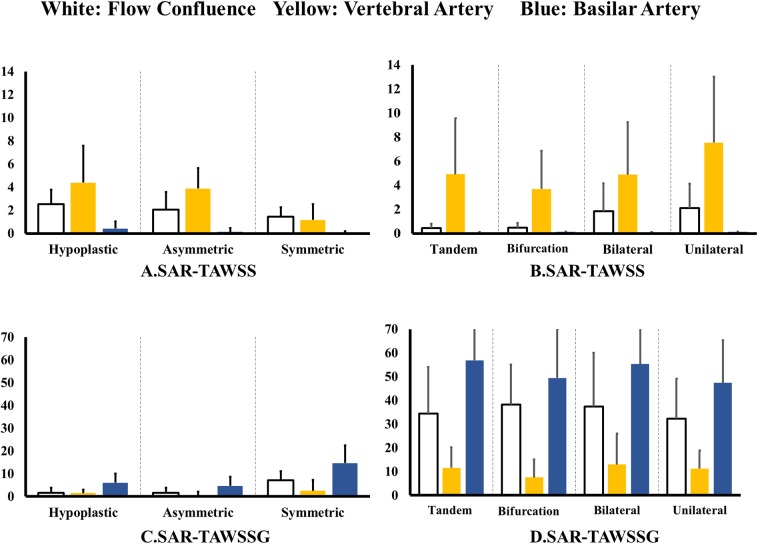
**(A,B)** SAR-TAWSS within flow confluence, vertebral artery, and basilar artery in **(A)** symmetric, asymmetric, and hypoplastic groups and **(B)** unilateral, bilateral, bifurcation, and tandem stenotic sub-groups and **(C,D)** SAR-TAWSSG within flow confluence, vertebral artery, and basilar artery in **(C)** symmetric, asymmetric, and hypoplastic groups and **(D)** unilateral, bilateral, bifurcation, and tandem stenotic sub-groups.

**Figure 5:**
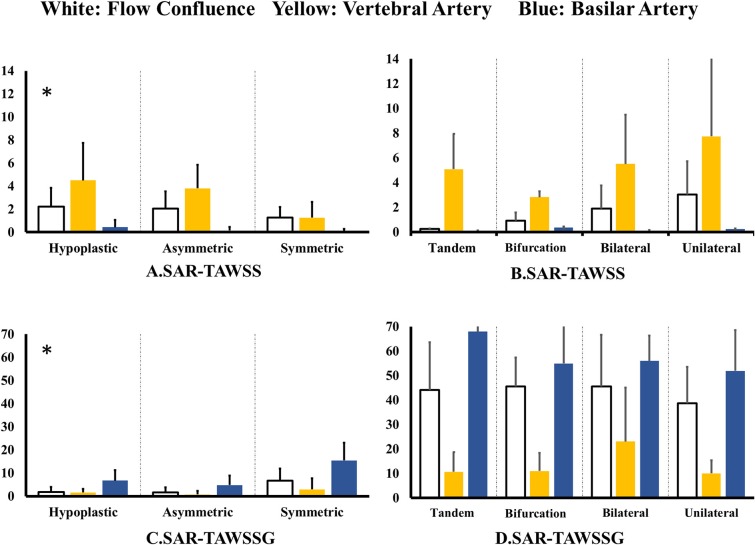
**(A,B)** SAR-TAWSS within flow confluence, vertebral artery, and basilar artery in **(A)** symmetric, asymmetric, and hypoplastic groups and **(B)** unilateral, bilateral, bifurcation, and tandem stenotic sub-groups and **(C,D)** SAR-TAWSSG within flow confluence, vertebral artery, and basilar artery in **(C)** symmetric, asymmetric, and hypoplastic groups and **(D)** unilateral, bilateral, bifurcation, and tandem stenotic sub-groups. All patients have TIAs or posterior infarctions in the unilateral, bilateral, bifurcation, and tandem stenotic sub-groups. All patients are normal in the symmetric, asymmetric, and hypoplastic groups.

Figure 4A shows SAR-TAWSS within flow confluence, vertebral artery, and basilar artery in symmetric, asymmetric, and hypoplastic groups and Figure 4B shows SAR-TAWSS in unilateral, bilateral, bifurcation, and tandem stenotic sub-groups. Figures 4C,D show SAR-TAWSSG corresponding to Figures 4A,B. SAR-OSI plots are neglected because of low values (<1%). Table 2 lists the corresponding hemodynamic parameters. SAR-TAWSS decreases, but SAR-TAWSSG increases in a sequence of vertebral artery, flow confluence, and basilar artery. SAR-TAWSSG values within flow confluence, vertebral artery, and basilar artery in the stenotic group are significantly higher than those in other groups despite relatively small difference of SAR-TAWSS values. Patients in the tandem stenotic sub-group have the highest TAWSSG and SAR-TAWSSG values. Figure 5 shows similar data as Figure 4, but patients without TIAs and posterior infarctions are taken out from unilateral, bilateral, bifurcation, and tandem stenotic sub-groups during the analysis while 13, 20, and 5 healthy patients are randomly selected from symmetric, asymmetric and hypoplastic groups, respectively. SAR-TAWSSG in Figure 5D is significantly higher than that in Figure 4D. Moreover, the conditional forward binary logistic regression shows SAR-TAWSSG (Wald test = 12.07, *p* < 0.001) as a better predictor of TIAs or posterior infarctions.

**Table 2:** Hemodynamic parameters within intracranial vertebrobasilar arteries in symmetric, asymmetric, hypoplastic, and stenotic groups as well as unilateral, bilateral, bifurcation, and tandem stenotic sub-groups.

**Groups**		**Symmetric (*N* = 74)**	**Asymmetric (*N* = 127)**	**Hypoplastic (*N* = 42)**	**Stenotic**			
					**Tandem (*N* = 37)**	**Bifurcation (11)**	**Bilateral (21)**	**Unilateral (31)**
TAWSS, dynes/cm^2^	Flow confluence	31.09 ± 11.04	21.27 ± 6.94	18.77 ± 5.72	56.63 ± 21.86	67.77 ± 28.07	53.64 ± 23.01	44.43 ± 12.36
	Vertebral artery	21.58 ± 8.47	17.1 ± 5.27	15.35 ± 5.77	32.65 ± 12.82	26.69 ± 9.61	27.59 ± 12.06	29.82 ± 16.12
	Basilar artery	43.08 ± 9.68	28.95 ± 9.36	28.99 ± 8.48	91.97 ± 31.83	70.62 ± 23.79	68.44 ± 20.95	59.87 ± 15.64
	Stenosis						115.14 ± 54.08	58.17 ± 20.06
TAWSSG, dynes/cm^3^	Flow confluence	248.75 ± 88.35	166.16 ± 55.53	166.13 ± 45.74	453.07 ± 174.86	542.16 ± 224.52	429.08 ± 184.06	355.43 ± 98.97
	Vertebral artery	172.68 ± 67.77	122.8 ± 42.19	138.77 ± 46.17	261.19 ± 102.54	213.50 ± 76.85	220.75 ± 96.51	238.59 ± 128.98
	Basilar artery	344.60 ± 77.46	226.97 ± 74.89	251.95 ± 67.90	735.72 ± 254.61	564.95 ± 190.30	547.5 ± 167.63	479.00 ± 125.09
	Stenosis						921.15 ± 432.66	465.35 ± 160.46

## Discussion

The present study carried out morphometric and hemodynamic analyses in intracranial vertebrobasilar arteries. The major findings of the study are reported as: (1) patients in the stenotic group have significantly higher incidence of TIAs or posterior infarctions than symmetric and asymmetric groups and patients in the tandem stenotic sub-group have the highest incidence; (2) patients in bilateral, bifurcation, and tandem stenotic sub-groups have significantly lower lumen size in the basilar artery than other groups; and (3) patients in the stenotic group have higher TAWSSG and SAR-TAWSSG than other groups and patients in the tandem stenotic sub-group have the highest values.

The independent risk factors for incidence and progression of intracranial artery stenosis include hypertension, diabetes mellitus, and so on (Bae et al., 2007; Huang et al., 2007). Intracranial arteries are predisposed to have proliferative fibrosis in intima or adventitia layers as compared with lipid infiltration in the atherosclerotic progression (Qureshi and Caplan, 2014). They are also susceptible to plaque instability given absence of an external elastic lamina (Masuoka et al., 2010), which often results in TIAs or posterior infarctions (Labadzhyan et al., 2011). Although patients in the stenotic group have relatively higher incidence of hypertension and diabetes mellitus, patients in other groups show the high incidence. Hence, there are other risk factors to explain the highest incidence of TIAs or posterior infarctions in the stenotic group, particularly the tandem stenotic sub-group.

Previous studies have shown that atherosclerotic plaques progress downstream to a stenosis, which is predisposed to form diffuse diseases (Huo et al., 2013a; Huang et al., 2016). Here, the lumen size along the normalized basilar artery of asymmetric and hypoplastic groups is similar to that in the symmetric group, but significantly higher than that in the stenotic group. This denotes higher incidence of diffuse diseases in the basilar artery of the stenotic group. The stenoses in vertebral arteries may be risk factors to stimulate the progression of diffuse diseases in the basilar artery. Furthermore, patients in the stenotic group were classified into the unilateral, bilateral, bifurcation, and tandem sub-groups. The lumen size along the normalized basilar artery of the unilateral sub-group, similar to that in symmetric and asymmetric groups, is significantly higher than the bilateral, bifurcation, and tandem sub-groups. Patients in the asymmetric group and unilateral stenotic sub-group have a dominant vertebral artery for posterior cerebral circulation, which showed similar flow patterns along the basilar artery to those in the symmetric group. In contrast, bilateral, bifurcation, and tandem stenoses in vertebral arteries could result in the disturbed flow patterns, e.g., flow vortex, stagnation point flow, and flow reversal that lead to low TAWSS, high OSI and TAWSSG in the distal basilar artery to the stenoses. Moreover, the conditional forward binary logistic regression analysis shows that two-sided vertebral artery stenoses or bifurcation stenoses are better risk factors for the development of diffuse basilar artery lesions than the symmetric, asymmetric, and hypoplastic vertebral arteries as well as the unilateral stenosis. Although it is required to investigate the mechanisms of different hemodynamic parameters for the growth of atherosclerotic plaques, bilateral, bifurcation, and tandem stenoses in vertebral arteries could still be risk factors to accelerate the development of diffuse diseases in the basilar artery.

### Hemodynamic Implications for TIAs or Posterior Infarctions

More than 50% plaque ruptures occurred at sites of diffuse artery lesions (Shah, 2003; Huo et al., 2013b; Arbab-Zadeh and Fuster, 2015) while others were at the upstream shoulder, neck, and downstream shoulder of a stenosis (Cheng et al., 2006; Toutouzas et al., 2015). Abnormal biomechanical stresses (e.g., low TAWSS, high OSI, and high TAWSSG) are believed to be critical risk factors for the development of vulnerable plaques as well as plaque ruptures (Chatzizisis et al., 2008; Seneviratne et al., 2013; Thondapu et al., 2016). For example, low TAWSS and high OSI can induce a sustained activation of a number of atherogenic genes in vascular endothelial cells (Chiu and Chien, 2011). The high low-density lipoprotein permeability and large sites of elevated permeability are associated with zones of elevated TAWSSG (Huo et al., 2007). All three abnormal parameters, low WSS, high OSI, and high WSSG, can result in the initiation and growth of atherosclerosis. However, there is the “low WSS-vs.-high WSSG” controversy for plaque ruptures (Seneviratne et al., 2013; Thondapu et al., 2016). The plaque rupture can be determined by the balance between plaque structural stress (PSS) and material strength, with plaque composition having a profound effect on PSS (Brown et al., 2016). The arterial regions that demonstrate high TAWSSG are generally associated with the higher PSS (Eshtehardi et al., 2017).

The present study shows significantly higher TAWSSG and SAR-TAWSSG values in the basilar arteries of the stenotic group than other groups. Patients in the tandem stenotic sub-group have higher value of TAWSSG and SAR-TAWSSG in the basilar artery and higher incidence of TIAs or posterior infarctions as compared with other sub-groups. Patients with TIAs or posterior infarctions from the tandem stenotic sub-group have the highest value of TAWSSG and SAR-TAWSSG in the basilar artery. Moreover, the conditional forward binary logistic regression analysis indicates SAR-TAWSSG as an independent risk factor for TIAs or posterior infarctions. A combination of high SAR-TAWSSG and diffuse diseases may stimulate high incidence of plaque ruptures in the basilar artery and hence results in TIAs or posterior infarctions, which still requires further investigations.

### Study Limitations

The retrospective study included 74, 127, 42, and 100 patients only in symmetric, asymmetric, hypoplastic, and stenotic groups, respectively. The sample size was relatively small. Morphometric and hemodynamic analyses only focused on the intracranial vertebrobasilar bifurcation that is comprised of left and right vertebral arteries and basilar artery and neglected other arteries (e.g., posterior cerebral artery, pontine perforators, and proximal segments of the vertebral artery). The following studies should carry out morphometric and hemodynamic analyses in the entire vertebrobasilar system of more patients. On the other hand, all patients have similar age (around 55 years) and are characterized in a single moment of their lives. The aortic pressure waves were determined by the scaling method, based on a representative pressure wave obtained from a previous study and the measured systolic and diastolic pressures in all patients. Here, we used a fixed pressure wave, omitting the influence of the specific pressure wave patterns from different patients. The scaling method can cause errors on the transient distribution of WSS, OSI, and WSSG in the intracranial vertebrobasilar bifurcation over a cardiac cycle albeit it has relatively small effects on the time-averaged values (Huo et al., 2009). The transient error needs to be considered in the computation. In the following studies, we plan to carry out a prospective study to investigate the development of diffuse basilar artery lesions, which includes as much morphometric and hemodynamic measurement in patients as possible.

## Conclusions

Morphometric and hemodynamic analyses were retrospectively carried out in the intracranial vertebrobasilar system of 343 patients. Patients in bilateral, bifurcation, and tandem stenotic sub-groups have significantly lower lumen size (i.e., higher incidence of diffuse diseases) in the basilar artery as compared with the unilateral stenotic sub-group as well as symmetric and asymmetric groups. Patients in the tandem stenotic sub-group have the highest SAR-TAWSSG and the highest incidence of TIAs or posterior infarctions. The high SAR-TAWSSG in the basilar artery is an independent risk factor for TIAs or posterior infarctions.

## Data Availability Statement

All datasets generated for this study are included in the article/Supplementary Material.

## Ethics Statement

This study was reviewed and approved by the Institutional Review Board (IRB) at the Affiliated Hospital of Hebei University, China.

## Author Contributions

XY, YE, and JL performed experiments. YF, TF, and WZ performed the theoretical analysis. YF and YH drafted the manuscript. YH and WT reviewed the manuscript. All authors approved it for publication.

### Conflict of Interest

The authors declare that the research was conducted in the absence of any commercial or financial relationships that could be construed as a potential conflict of interest.

## Nomenclature

1. **SAR-TAWSS within the vertebral arteries**: surface area ratio of low TAWSS ($=\frac{Surface\;area_{TAWSS\leq 4\;dynes•cm^{-2}}}{Total\;surface\;area\;of\;the\;vertebral\;arteries\;}\times 100\%$) within the vertebral arteries. Surface area of TAWSS ≤ 4 dynes/cm^2^ refers to the disease-prone site (Malek et al., 1999; Kleinstreuer et al., 2001).
2. **SAR-TAWSS within the flow confluence**: surface area ratio of low TAWSS ($=\frac{Surface\;area_{TAWSS\leq 4\;dynes•cm^{-2}}}{Surface\;area\;of\;flow\;confluence}\times 100\%$) in the flow confluence. “Surface area of flow confluence” refers to the surface area of flow confluence in Figure 1.
3. **SAR-TAWSS within the basilar artery**: surface area ratio of low TAWSS ($=\frac{Surface\;area_{TAWSS\leq 4\;dynes•cm^{-2}}}{Total\;surface\;area\;of\;the\;basilar\;artery}\times 100\%$) within the basilar artery.
4. **SAR-OSI within the vertebral arteries**: surface area ratio of high OSI ($=\frac{Surface\;area_{OSI\geq 0.15}}{Total\;surface\;area\;of\;the\;vertebral\;arteries}\times 100\%$) within the vertebral arteries. Surface area of OSI ≥ 0.15 refers to the disease-prone site (Nordgaard et al., 2010; Huo et al., 2013a).
5. **SAR-OSI within the flow confluence**: surface area ratio of high OSI ($=\frac{Surface\;area_{OSI\geq 0.15}}{Surface\;area\;of\;flow\;confluence\;}\times 100\%$) within the flow confluence.
6. **SAR-OSI in the basilar artery**: surface area ratio of high OSI ($=\frac{Surface\;area_{OSI\geq 0.15}}{Total\;surface\;area\;of\;the\;basilar\;artery\;}\times 100\%$) within the basilar artery.
7. **SAR-TAWSSG within the vertebral arteries**: surface area ratio of high TAWSSG ($=\frac{Surface\;area_{TAWSSG\geq 500\;dynes•cm^{-3}}}{Total\;surface\;area\;of\;the\;vertebral\;arteries}\times 100\%$) within the vertebral arteries. Surface area of TAWSSG ≥ 500 dynes/cm^3^ refers to the disease-prone site (Fan et al., 2016).
8. **SAR-TAWSSG in the flow confluence**: surface area ratio of high TAWSSG ($=\frac{Surface\;area_{TAWSSG\geq 500\;dynes•cm^{-3}}}{Surface\;area\;of\;flow\;confluence}\times 100\%$) within the flow confluence.
9. **SAR-TAWSSG within the basilar artery**: surface area ratio of high TAWSSG ($=\frac{Surface\;area_{TAWSSG\geq 500\;dynes•cm^{-3}}}{Total\;surface\;area\;of\;the\;basilar\;artery\;}\times 100\%$) within the basilar artery.
